# Disrespect and abuse during childbirth in East Hararghe Zone public health facilities, eastern Ethiopia: a cross-sectional study

**DOI:** 10.3389/fgwh.2023.1237098

**Published:** 2023-11-30

**Authors:** Ahmedin Aliyi Usso, Hassen Abdi Adem, Addisu Alemu, Aminu Mohammed

**Affiliations:** ^1^ School of Nursing and Midwifery, College of Health and Medical Sciences, Haramaya University, Harar, Ethiopia; ^2^ School of Public Health, College of Health and Medical Sciences, Haramaya University, Harar, Ethiopia; ^3^ Department of Midwifery, College of Medicine and Health Sciences, Dire Dawa University, Dire Dawa, Ethiopia

**Keywords:** disrespect and abuse, respectful maternity care, childbirth, health facilities, Ethiopia

## Abstract

**Background:**

Compassionate and respectful maternity care during childbirth has been identified as a potential strategy to prevent and reduce maternal mortality and morbidity. Despite its importance, there is a paucity of information on the level of disrespect and abuse meted out to mothers in eastern Ethiopia. This study assesses the level of disrespect and abuse suffered by women during childbirth, and the associated factors, in public health facilities in the rural East Hararghe Zone in eastern Ethiopia.

**Methods:**

A cross-sectional study was conducted among 530 women who gave birth in 20 public health facilities in the East Hararghe Zone during the period between 1 April and 30 April 2020. Data were collected using a validated questionnaire. Bivariable and multivariable binary logistic regression analyses were employed to identify the factors associated with disrespect and abuse during childbirth. Adjusted odds ratio (AOR) (95% CI) was used to report this association, and statistical significance was set at *P* < 0.05.

**Results:**

Overall, 77% (95% CI: 73%–81%) of women reported at least one type of disrespect and abuse during childbirth in the East Hararghe Zone public health facilities. In this study, factors such as households having an average monthly income of below 57.22 USD (AOR = 2.29, 95% CI: 1.41–3.71), mothers residing at more than 30 min away from a nearby health facility (AOR = 2.10, 95% CI: 1.30–3.39), those not receiving antenatal care (AOR = 4.29, 95% CI: 2.17–8.52), and those giving birth during nighttime (AOR = 2.16, 95% CI: 1.37–3.41) were associated with at least one type of disrespect and abuse during childbirth.

**Conclusion:**

More than three in every four women who gave birth in the East Hararghe Zone public health facilities were disrespected and abused during childbirth. Encouraging all pregnant women to pay attention to antenatal care visits and improving the quality of healthcare service during nighttime in all health facilities will be essential for preventing and reducing disrespect and abuse and its negative consequences.

## Introduction

Respectful maternity care is a basic human right for every woman and should be provided in every health facility to any woman in a way that protects her dignity, privacy, and confidentiality and safeguards her from harm and mistreatment while ensuring informed choice and continuity of healthcare (1, 2). It is, in fact, an element of the quality of maternity care (3). Disrespect and abuse constitute any form of inhumane behavior applied in the care of women during childbirth. It is a violation of women's human rights and an aspect of poor quality of care, which negatively influences the coverage of institutional birth (1, 4) and indirectly increases the risks of maternal and neonatal mortality worldwide, particularly in sub-Saharan African (SSA) countries.

The proportion of births attended by skilled professionals increased in higher-income countries in the last decade; yet, the coverage of skilled birth attendance remained low in low- and middle-income countries (LMIC) (5, 6). For instance, in 2018, the proportion of skilled birth attendance was 81% globally, almost 100% in higher-income countries, 82% in Southeast Asia, 60% in SSA (6), and 48% in Ethiopia (7). Providing poor quality service and mistreatment by providers in health facilities may contribute to a low coverage of skilled birth attendance (8, 9).

Maternal mortality is a major public health problem in developing countries, particularly in SSA (10). Ensuring skilled birth attendance during childbirth is the basic strategy for preventing and reducing maternal mortality in these countries (11–13). According to data provided in 2020, an estimated 287,000 maternal deaths occurred worldwide every year because of birth-related complications; 95% of these deaths occurred in LMIC, and more than 70% of these deaths occurred at a ratio of 545 maternal deaths per 100,000 live births in SSA; 267 maternal deaths per 100,000 live births occurred in Ethiopia in 2020 (14). These higher rates of maternal mortality were associated with a low rate of skilled birth attendance in these countries (6, 10).

The Ethiopian government’s health-sector transformation based on the Sustainable Development Goals (SDGs) plans to reduce the maternal mortality rate to 70 per 100,000 by 2030 (15, 16), which can be achieved only by improving the quality of maternal healthcare services by ensuring that respectful maternity care service is provided to all women at every level of contact with health facilities (17). Improving respectful maternity care has already been identified as a potential strategy to prevent and reduce maternal mortality, which can be achieved only by improving the coverage of skilled birth attendance (3, 18).

The burden of disrespect and abuse was noted to be higher in developing countries (9), being much higher in SSA, including Ethiopia, ranging from 20% to 98% in SSA (9, 19–21) and 40%–92.5% in Ethiopia (22–24).

Sociodemographic factors such as the age of women, their area of residence, marital status, educational status, wealth status, and their HIV seropositive status are those that influence the level of disrespect and abuse caused to mothers during childbirth in health facilities (22, 25, 26).

Despite the extent and magnitude of the problem, little is known about the level of disrespect and abuse suffered by women during childbirth in health facilities in Ethiopia. A few previous studies were conducted among women from only their urban residences (22, 24) and hospitals (23, 27), which cannot sufficiently address the main problem of disrespect and abuse in a public health facility. Overall, there is limited information on disrespect and abuse during childbirth in rural eastern Ethiopia. Therefore, this study assesses the level of disrespect and abuse meted out to women during childbirth in a specific setting, that is, in public health facilities in the East Hararghe Zone of eastern Ethiopia.

## Methods and materials

### Study design and setting

This is an institution-based cross-sectional study conducted among 530 women who gave birth in randomly selected public health facilities in the East Hararghe Zone during the period between 1 April 2020 and 30 April 2020. East Hararghe Zone is one of the 24 zones of the Oromia region located in eastern Ethiopia. Administratively, the East Hararghe Zone separates into 25 districts and four towns and has an estimated total population of 3,821,021, with 845,592 women in the reproductive age group and 132,590 pregnant women, as reported in the year 2019. According to the zonal health office annual report 2019, there are five hospitals and 120 health centers. However, there were 85 accessible public health facilities (five hospitals and 80 health centers) in the zone during the study period. Twenty public health facilities (two hospitals and 18 health centers) were selected using a simple random sampling method.

### Study participants

All women who give birth in all public health facilities in the East Hararghe Zone comprised the source population. Women who gave birth in randomly selected public health facilities in the zone during the study period constituted the study population. Women who gave birth in randomly selected public health facilities during the data collection period and who were 18 years old and above were included in the study. Women who were critically ill and unable to respond to interviews, who gave birth through cesarean section, and who were referred to other health facilities were excluded from the study.

### Sample size determination and sampling procedures

The sample size was calculated by using Epi Info version 7.1 considering the assumptions for single (prevalence of disrespect and abuse) and double (predictor factors of disrespect and abuse) population proportion formulas. Accordingly, the maximum sample size (*n* = 542) was obtained from a single population proportion formula, considering a 78.6% proportion of mothers who suffered disrespect and abuse, with the following assumptions: a confidence level of 95%, a margin of error of 5%, and a proportion of women who experienced disrespect and abuse (78.6%) in Addis Ababa, Ethiopia (22), using a design effect of 2 and a 5% non-response rate. Study participants were selected using a multistage stratified sampling technique. We stratified facilities as hospitals and health centers, and two hospitals out of five and 18 health centers out of 80 were selected using a simple random sampling technique. Then, the estimated sample size was proportionally allocated to each facility (based on the average birth attendance flow in the previous 3 months). Eligible women were selected consecutively from each selected health facility. After obtaining written informed consent from them, an exit interview was conducted during the time of discharge.

### Data collection tool and measurement

Data were collected using a pretested structured questionnaire adapted from validated scales and relevant published literature (9, 22, 23, 28–30). We pretested the adapted questionnaire on 5% of the total sample (27 women) to check its validity in a separate non-selected public health facility (Ugas Health Center) in the East Hararghe Zone, and changes were made accordingly before the actual data collection process. The questionnaire consisted of sociodemographic characteristics, reproductive health conditions, healthcare-related factors, and disrespect and abuse during childbirth. Disrespect and abuse were measured using a framework developed by Bowser and Hill (29) and categorized into seven domains (physical abuse, non-confidential care, non-informed consent care, non-dignified care, abandonment of care, discrimination, and detention). Each category had more than one verification criteria with dichotomized (yes/no) responses, and a total of 24 verification criteria were used to measure these seven categories. The women were considered to have been disrespected and abused if they reported “Yes” to at least one verification criterion of these categories (22, 28).

### Data quality control

The quality of the data was maintained using standard questionnaires adapted from validated scales and relevant published literature. The questionnaire, first prepared in English, was translated into the local language (Afan Oromo) and back to English by two experts having a good command of both languages. Twelve diploma nurses who were not employees of the selected health facilities were trained to collect the data under the supervision of five supervisors after receiving training for one day on the objectives of the study and the data collection technique to be employed.

### Data processing and analysis

After checking for completeness, the data were entered into EpiData version 3.1 and analyzed using SPSS version 24. Descriptive statistics such as the frequency and measure of central tendency were used to characterize the study participants. Before conducting the analysis, the internal consistency of the items was checked for the presence of composite index variables using reliability analysis (Cronbach *α *= 0.92). Bivariable and multivariable logistic regression analyses were conducted to identify the factors associated with disrespect and abuse during childbirth. Multivariable binary logistic regression analyses were fitted to determine significant risk factors, and the Hosmer and Lemeshow goodness of fit test at a *p*-value > 0.05 was used to confirm the overall adequacy of the model. Adjusted odds ratio (AOR) with its 95% confidence interval (CI) was used to report the strength of association and the statistical significance declared at a *p*-value < 0.05.

## Results

### Sociodemographic characteristics

A total of 542 eligible women who gave birth in selected public health facilities were invited to participate in the study, and 530 of them enrolled in the study with a 97.8% response rate. The mean age of the participants was 26.1 (±5.4) years. The majority, 402 (75.8%) of the respondents, were from rural residences, and almost all (97.5%) women were married. With regard to occupational status, most (88.3%) of the participants were housewives, followed by 32 (6.0%) government employees and 16 (3.0%) merchants. More than half (53.5%) of the participants, had no formal education, followed by 195 (36.8%) primary school and 51 (9.6%) secondary school education and above. With regard to parity status of the participants, approximately 131 (24.3%) of them were grand multiparous mothers. In terms of average monthly income, a majority (51.3%) of the participants had an average monthly income of less than 57.22 United States Dollar (USD) (Table 1).

**Table 1:** Sociodemographic characteristics of women who gave birth in East Hararghe Zone public health facilities, eastern Ethiopia, 2020 (*n* = 530)**.**

Variables	Categories	Frequency	Percent
Type of health facility	Health center	434	81.9
	Hospital	96	18.1
Residence area	Urban	128	24.2
	Rural	402	75.8
Age (in years)	18–24	176	33.2
	25–34	297	56.0
	≥35	57	10.8
Marital status	Married	517	97.5
	Single	13	2.5
Religion	Muslim	471	88.9
	Orthodox	44	8.3
	Protestant	15	2.8
Ethnicity	Oromo	458	86.4
	Amhara	34	6.4
	Guraghe	18	3.4
	Other^a^	20	3.8
Women's occupation	Housewife	468	88.3
	Merchant	16	3.0
	Employee	32	6.0
	Other^b^	35	2.7
Partner's occupation	Farmer	387	74.6
	Merchant	50	9.6
	Employee	60	11.6
	Other^b^	21	4.2
Education of women	No formal education	284	53.5
	Primary education	195	36.8
	Secondary and above	51	9.6
Education of partner	No formal education	203	39.3
	Primary education	237	45.8
	Secondary and above	77	14.9
Average monthly income (in USD)^c^	≤57.22	272	51.3
	>57.22	258	48.7
Time to reach nearby public health facility (min)	≤30	156	29.4
	>30	374	70.6

^a^ Somali or Orgoba.

^b^ Daily laborers or students; USD, United States Dollar.

^c^ 1 USD = 34.95ETB during the study period.

### Reproductive health and healthcare service characteristics

The means (+SD) of gravidity and parity were 3.7 (±2.5) and 3.4 (±2.3), respectively. Approximately 399 (75.3%) women had a parity status less than or equal to four childbirths. Less than one-fourth (21.7%) of women experienced at least one abortion. The mean (+SD) of alive children was 3.2 (±2.1). The majority (83.2%) of the participants gave birth through spontaneous vaginal delivery (SVD), and more than nine in every 10 childbirths (93.8%) were delivered alive. With regard to the gender of the main birth attendance, half (50.4%) of the participants were attended by females. Approximately 70.6% of participants traveled for more than 30 min on foot to reach the nearby health facility. A majority (70.4%) of the women attended antenatal care (ANC) visit at least once. Seven out of ten women (69.6%) gave birth during nighttime (Table 2).

**Table 2:** Reproductive characteristics of women who gave birth in East Hararghe Zone public health facilities, eastern Ethiopia, 2020 (*n* = 530).

Variables	Categories	Frequency	Percent
Parity	≤4	399	75.3
	>4	131	24.7
Visited ANC facility	Yes	373	70.4
	No	157	29.6
Mode of delivery	SVD	441	83.2
	Not SVD^a^	89	16.8
Birth outcome (of newborn)	Alive	497	93.8
	Stillbirth	33	6.2
Gender of main birth attendant	Male	263	49.6
	Female	267	50.4
Delivery time	Daytime	161	30.4
	Nighttime	369	69.9
Current pregnancy intention	Planned	415	78.3
	Unplanned	115	21.7

^a^ Induced vaginal delivery and/or assisted vaginal delivery.

### Disrespect and abuse during childbirth

In this study, the magnitude of disrespect and abuse experienced by women during childbirth in a public health facility could be gauged from the actual figures; the overall rate was 77.0% (95% CI: 73%–81%). Based on the type of health facilities, the rates of disrespect and abuse were 72.0% and 78.0% among women who gave birth in hospitals and health centers, respectively. Among disrespected and abused women, the category of non-informed consent care was the most prevalent one, with the figure standing at 259 (48.9%). Under these categories, the most commonly reported type of disrespect and abuse was that the provider did not introduce themselves to the woman and her companion, 259 (48.9%). The second most reported category of disrespect and abuse was non-confidential care, 252 (47.5%). Among the verification types of non-confidential care, 177 (33.4%) of women reported that care providers did not use drape or cover to protect their privacy. The third most reported category was physical abuse, reported by 142 (26.8%) women, followed by non-dignified care, 135 (25.5%), abandonment care, 53 (10.0%), discrimination, 29 (5.5%), and detention, 13 (2.5%) (Table 3).

**Table 3:** Categories of disrespect and abuse during childbirth by verification criteria among women who gave birth in East Hararghe Zone public health facilities, eastern Ethiopia, 2020 (*n* = 530).

Reported types of disrespect and abuse during childbirth	Frequency	Percentage
Physical abuse	**142**	**26.8**
Health provider physically hit or slapped the mother	99	18.7
Health provider verbally insulted the mother	120	22.6
Care provider separated the mother from the baby without indication	50	9.4
Supportive staff insulted the mother and her companion	50	9.4
Provider denied the mother food or fluid without indication	89	16.8
Provider did not permit the mother to choose a position for birth	96	19.1
Non-confidential care	**252**	**47.5**
Care providers did not use covering to protect the mother’s privacy	177	33.4
Providers discussed the mother's private parts in a way others could hear	105	19.8
Non-informed consent care	**259**	**48.9**
Provider did not introduce him/herself to the mother	259	48.9
Providers did not explain the findings to the mother	67	12.6
Providers did not explain to the mother what was done or what to expect	67	12.6
Providers did not encourage the mother to ask questions	86	16.2
Provider did not respond to the mother's question with politeness	99	18.7
Provider did not obtain consent before a procedure	233	44.0
Non-dignified care	**135**	**25.5**
Health providers shouted at or intimidated the mother	135	25.5
Health providers made negative comments about the mother	111	20.9
Providers did not allow the mother's companion into the delivery room	115	21.5
Abandonment of care	**53**	**10.0**
Providers ignored or abandoned the mother when called for help	53	10
The mother gave birth alone because providers were not present	11	2.1
Discrimination	**29**	**5.5**
Provider discriminated against the mother on educational or economic status	29	5.5
Provider discriminated against the mother on residential area	16	3.0
Providers discriminated against the mother because of her age	10	1.9
Detention in health facilities	**13**	**2.5**
The mother's discharge was postponed until facility bills were paid	9	1.7
The mother was detained in the health facility because of damage to property	4	0.8

The bold value indicates the overall prevalence of each category of disrespect and abuse, while the no-bold value indicates the frequency of items under respective categories.

### Factors associated with disrespect and abuse during childbirth

In the bivariable analysis, the area of residence, age of women, average monthly income, distance from a nearby public health facility, parity, visiting ANC facilities, and time of delivery were the factors associated with disrespect and abuse during childbirth.

In the multivariable analysis, average monthly income, distance from a nearby public health facility, parity, visiting ANC facilities, and time of delivery were the factors independently associated with disrespect and abuse during childbirth. Women who received an average monthly income of less than 57.22 USD were two times more likely to be disrespected and abused (AOR = 2.29, 95% CI: 1.41–3.71) than those who had an average monthly income greater than or equal to 57.22 USD. Women living in residences at distances greater than 30 min from nearby health facilities were two times (AOR = 2.10 95% CI: 1.30–3.39) more likely to experience disrespect and abuse during childbirth at these facilities than those who lived less than or equal to 30 min away from the health facilities. Women with a parity status of less than or equal to four faced an approximately 1.7 time increase in the risk of disrespect and abuse than those with a parity status of more than four childbirths (AOR = 1.70, 95% CI: 1.02–2.84). Women who never paid a visit to an ANC facility during their current pregnancy were four times (AOR = 4.29, 95% CI: 2.17–8.52) more likely to be disrespected and abused during childbirth than their visiting counterparts. Women who delivered during nighttime were approximately two times more likely to be disrespected and abused (AOR = 2.16, 95% CI: 1.37–3.41) than those who delivered during daytime (Table 4).

**Table 4:** Factors associated with disrespect and abuse during childbirth among women who gave birth in East Hararghe Zone public health facilities, eastern Ethiopia, 2020 (*n* = 530).

Variables	Categories	Disrespected and abused		COR (95% CI)	AOR (95% CI)
		Yes, *n* (%)	No, *n* (%)		
Residential area	Urban	77 (60.2)	51 (39.8)	1	1
	Rural	331 (82.3)	71 (17.7)	**3.09 (1.99–4.78)** ***	1.33 (0.65–2.72)
Age (in year)	18–24	146 (83.0)	30 (17.0)	**2.84 (1.46–5.53)** **	1.78 (0.71–4.42)
	25–34	226 (76.1)	71 (23.9)	**1.86 (1.02–3.38)** *	1.58 (0.74–3.34)
	≥35	36 (63.2)	21 (36.8)	1	1
Monthly income (in USD)	<57.22	238 (87.5)	34 (12.5)	**3.62 (2.33–5.64)** ***	**2.29 (1.41–3.71)** ***
	≥57.22	170 (65.9)	88 (34.1)	1	1
Distance from nearby health facility (min)	≤30	94 (60.3)	62 (39.7)	1	1
	>30	314 (84.0)	60 (16.0)	**3.45 (2.26–5.27)** ***	**2.10 (1.30–3.39)** **
Types of health facility	Health center	339 (78.1)	95 (21.9)	1.40 (0.85–2.30)	1.07 (0.60–1.92)
	Hospital	69 (71.9)	27 (28.1)	1	1
Parity	≤4	313 (78.4)	86 (21.6)	1.38 (0.88–2.17)	**1.70 (1.02–2.84)** *
	>4	95 (72.5)	36 (27.5)	1	1
Mode of delivery	SVD	338 (76.6)	103 (23.4)	1.12 (0.65–1.95)	0.88 (0.46–1.70)
	No SVD^a^	70 (78.7)	19 (21.3)	1	1
Birth outcome	Alive	380 (76.5)	117 (23.5)	1	1
	Dead	28 (84.8)	5 (15.2)	1.72 (0.65–4.57)	1.01 (0.32–3.15)
Antenatal care attendance	Yes	262 (70.2)	111 (29.8)	1	1
	No	146 (93.0)	11 (7.0)	**5.62 (2.93–10.79)** ***	**4.29 (2.17–8.52)** ***
Delivery time	Daytime	101 (62.7)	60 (37.3)	1	1
	Nighttime	307 (83.2)	62 (16.8)	**2.94 (1.93–4.48)** ***	**2.16 (1.37–3.41)** ***
Gender of main birth attendance	Male	203 (77.2)	60 (22.8)	1.02 (0.68–1.53)	1.15 (0.73–1.81)
	Female	205 (76.8)	62 (23.2)	1	1

COR, crude odds ratio.

The bold value indicates for variable having *p*-value < 0.05, while the non-bold value indicates variable having *p*-value > 0.05.

^a^ Induced vaginal delivery and/or assisted vaginal delivery.

* *p* < 0.05.

** *p* < 0.01.

*** *p* < 0.001.

## Discussion

This study assessed the magnitude and associated factors of disrespect and abuse during childbirth among women who gave birth in East Hararghe Zone public health facilities in eastern Ethiopia. We found that more than three in every four women who gave birth in these facilities were disrespected and abused during childbirth. Low average monthly incomes, living at far-off distances from nearby health facilities, low parity, a lack of ANC attendance, and delivery during nighttime were independent predictors of disrespect and abuse during childbirth.

This study found that the rate of disrespect and abuse suffered by women was 77.0% (95% CI: 73.0%–81.0%), which is in line with those found in studies conducted in Addis Ababa, Ethiopia (78%) (22) and western Ethiopia (74.8%) (28). However, this rate was higher than those found in studies conducted in Bale, Ethiopia (37.5%) (23); Addis Ababa, Ethiopia 17.5% (31); Bahirdar, Ethiopia (67.1%) (24); Kenya (20%) (21); and Tanzania (15%) (30). These differential rates might be attributed to sociodemographic differences: almost all study participants were urban residents (24, 31), compared with three-fourths of our study participants who were rural residents. In addition, the difference in the type of health facilities might be another possible explanation for the differential rates because our study included both hospitals and health centers, while a previous study conducted in Bale excluded health centers. Moreover, the sociocultural status of women, their general attitude toward maternity care, and infrastructural problems in health facilities may increase the rate of prevalence. For instance, the number of institutional deliveries has increased in rural eastern Ethiopia without corresponding increases in infrastructure, staff, and supplies, which compromises the parameters of respectful maternity care.

Conversely, the rate of disrespect and abuse suffered by women was lower than those found in the studies conducted in Jimma, southern Ethiopia (91.7%) (27), Malawi (93.7%) (32), Nigeria (98%) (9), and Pakistan (97%) (33). This variation might be a result of the different verification criteria used to measure disrespect and abuse; 24 verification items were used for seven domains of disrespect and abuse in our study, while comparable studies used 48 or more verification criteria (34, 35), which might result in over-reporting. In addition, this difference might be attributed to methodological differences; in our study, we used face-to-face interviews during data collection, while a comparable study used direct observation of labor and delivery to assess the prevalence of disrespect and abuse (32).

This study showed that women who received an average monthly income of less than 57.22 USD were two times more likely to be disrespected and abused than those who had an income of more than or equal to 57.22 USD. The economic status of a woman is a significant barrier to compassionate and respectful maternity care in public health facilities. This implies that poor women were more likely to experience disrespect and abuse during childbirth in health facilities (36). This finding was in agreement with that of other studies conducted in Ethiopia (22, 24, 34, 28) and might be attributed to providers prioritizing women with higher incomes over those with lower incomes; affluent women received more timely care than poor women, regardless of the severity of their medical condition (28, 37).

Women living at greater distances from public health facilities were more likely to be disrespected and abused during childbirth. This could be attributed to the fact that such women had comparatively limited knowledge, involvement, and decision-making abilities, which makes them passive and powerless in the care they receive. Hence, such category of women not receiving respectful maternity care during childbirth is a problem that needs to be addressed. It is important to improve the coverage of both the quality of healthcare and the access of healthcare services to ensure respectful maternity care.

The parity status of less than or equal to four childbirths was approximately 1.7 times more likely to increase the risks of disrespect and abuse during childbirth in health facilities. This finding was in agreement with that in the study conducted in Kenya (21); multiparous women are more familiar with maternity care services provided in health facilities and may ask for missed services during childbirth.

This study indicated that women who did not attend ANC visits during pregnancy were four times more likely to experience disrespect and abuse during childbirth. This finding was supported by that in the studies conducted in Bale, Ethiopia (23), and Bahirdar, Ethiopia (38). Good maternity care utilization habits could improve women's awareness about the health system, which would empower them to defend themselves from disrespect and abuse during childbirth. In addition, availing of ANC facilities in a timely manner might help create a strong bond between women and care providers. This means that providing access to ANC facilities to all pregnant women in health facilities is important for reducing mistreatment during childbirth.

Women who delivered during nighttime were approximately two times more likely to be disrespected and abused than those who delivered during daytime. This finding was supported by those in studies conducted in Central Ethiopia (39), Northwest Ethiopia (40), and Kenya (21), which indicated that women who delivered during the day were more likely to receive respectful maternity care than those who delivered during the night. This could be attributed to an inadequate number of care providers assigned during nighttime, resulting in healthcare providers becoming tired because of work overload (40). On the other hand, the trend of providing poor quality maternity care during nighttime might be associated with low staff numbers assigned to obstetric cases during nightshift (41). Infrastructural problems such as power interruption might be another challenge for women receiving respectful maternity care during nighttime (42, 43). It is a worrisome fact that women in the labor ward during nighttime were more disrespected and abused than their daytime counterparts. It is important to put in place mechanisms to ensure respectful maternity care during nighttime, which can be achieved by increasing the number of care providers assigned and considering nighttime supervision in all health facilities.

The strength of this study is that recall bias was minimized because of an exit interview conducted during discharge. Moreover, hospitals and health centers that provide women with different maternity care services were included in the study and could be generalized to eastern Ethiopia and beyond. However, disrespect and abuse were measured using interviews alone, and this might be considered a limitation of the study; because of labor pain, the women in labor were less stable when providing care, and the reported extent may deviate from the actual value.

## Conclusion

More than three in every four women who delivered in public health facilities in the East Hararghe Zone reported at least one type of disrespect and abuse during childbirth. Average monthly income, distance from nearby health facilities, attendance at ANC facilities, and nighttime delivery were the factors significantly associated with disrespect and abuse during childbirth. Encouraging all pregnant women to pay attention to ANC visits and improving the quality of healthcare service provided in all health facilities by assigning adequate staff and fulfilling supplies required during the night are essential for reducing and preventing disrespect and abuse. In addition, specific strategies and interventions should be designed to ensure equitable access to quality maternity care during childbirth for women living at far-off distances from health facilities. Furthermore, we recommend that future researchers conduct further studies using direct observation during the provision of maternity care service.

## Data Availability

The original contributions presented in the study are included in the article/Supplementary Material, and further inquiries can be directed to the corresponding author.
